# Secreted Peptide PIP1 Induces Stomatal Closure by Activation of Guard Cell Anion Channels in *Arabidopsis*

**DOI:** 10.3389/fpls.2020.01029

**Published:** 2020-07-08

**Authors:** Jianlin Shen, Wenzhu Diao, Linfang Zhang, Biswa R. Acharya, Mei Wang, Xiangyu Zhao, Donghua Chen, Wei Zhang

**Affiliations:** ^1^Key Laboratory of Plant Development and Environmental Adaption Biology, Ministry of Education, School of Life Science, Shandong University, Qingdao, China; ^2^College of Natural and Agricultural Sciences, University of California Riverside, Riverside, CA, United States; ^3^State Key Laboratory of Crop Biology, College of Life Sciences, Shandong Agricultural University, Tai'an, China

**Keywords:** guard cells, secreted peptide, PIP1, stomatal closure, anion channel

## Abstract

Plant stomata which consist of a pair of guard cells, are not only finely controlled to balance water loss as transpiration and CO_2_ absorption for photosynthesis, but also serve as the major sites to defend against pathogen attack, thus allowing plants to respond appropriately to abiotic and biotic stress conditions. The regulatory signaling network for stomatal movement is complex in nature, and plant peptides have been shown to be involved in signaling processes. Arabidopsis secreted peptide PIP1 was previously identified as an endogenous elicitor, which induced immune response through its receptor, RLK7. PIP1-RLK7 can activate stomatal immunity against the bacterial strain *Pst* DC3118. However, the molecular mechanism of PIP1 in stomatal regulation is still unclear and additional new factors need to be discovered. In this study, we further clarified that PIP1 could function as an important regulator in the induction of stomatal closure. The results showed that PIP1 could promote stomata to close in a certain range of concentrations and response time. In addition, we uncovered that PIP1-RLK7 signaling regulated stomatal response by activating S-type anion channel SLAC1. PIP1-induced stomatal closure was impaired in *bak1*, *mpk3*, and *mpk6* mutants, indicating that *BAK1* and *MPK3/MPK6* were required for PIP1-regulated stomatal movement. Our research further deciphered that *OST1* which acts as an essential ABA-signaling component, also played a role in PIP1-induced stomatal closure. In addition, ROS participated in PIP1-induced stomatal closure and PIP1 could activate Ca^2+^ permeable channels. In conclusion, we reveal the role of peptide PIP1 in triggering stomatal closure and the possible mechanism of PIP1 in the regulation of stomatal apertures. Our findings improve the understanding of the role of PIP1 in stomatal regulation and immune response.

## Introduction

Plant stomatal pores are formed by pairs of specialized epidermal guard cells and serve as major gateways to modulate gas exchange for photosynthesis and transpirational water loss (Bergmann and Sack, 2007). Stomata are also the major entry sites for different kinds of pathogens. Plants frequently suffer from various abiotic and biotic stresses during their life cycle, and stomata play an important role in allowing plants to respond appropriately to diverse environmental stimuli or defend against pathogen attack (Melotto et al., 2006; Kim et al., 2010; Blatt et al., 2017). The regulatory signaling pathway of stomatal movement is complex and a lot of elements can influence stomatal pore apertures, such as CO_2_, light, humidity, phytohormones, pathogens (Speth et al., 2009; Kollist et al., 2014; Murata et al., 2015). Among these factors, plant stress hormone abscisic acid (ABA) signaling has been studied extensively and shown to play a vital role in the regulation of stomatal movement (Wang et al., 2001; Raghavendra et al., 2010; Lee et al., 2013). ABA can trigger the activation of guard cell anion channels (e.g. SLAC1) and can result in the efflux of anions, which in turn reduces the turgor of guard cells to promote stomatal closure (Li et al., 2000; Cutler et al., 2010; Okamoto et al., 2013; Merilo et al., 2015). In addition, the cytosol reactive oxygen species (ROS) and Ca^2+^ are important second messengers which are elevated in response to ABA that in turn function as positive regulators in ABA-mediated stomatal closure (Pei et al., 2000; Kim et al., 2010; Suzuki et al., 2011; Singh et al., 2017).

There are numerous small signaling peptides have been discovered in plants and most of them do not have known function. Primarily, small signaling peptides have been found to play roles in plant growth and development (Wang and Fiers, 2010; Murphy et al., 2012; Czyzewicz et al., 2013; Dong et al., 2019). Additional findings also implicate that small signaling peptides can play roles in regulating stomatal movement and can response to abiotic or biotic stress (Wang et al., 2016; Takahashi et al., 2018; Yu et al., 2018; Zheng et al., 2018; Qu et al., 2019). flg22 is a 22-amino acid peptide, which is derived from the bacterial flagellin protein (Monaghan and Zipfel, 2012). In *Arabidopsis*, flg22 can be recognized by the receptor kinase FLS2 in plasma membrane and then FLS2 interacts with BRI1-associated kinase 1 (BAK1) to form an active complex by phosphorylation (Chinchilla et al., 2007; Sun et al., 2013). Besides, another receptor-like kinase, BOTRYTIS-INDUCED KINASE 1 (BIK1), is a direct substrate of the FLS2-BAK1 complex which phosphorylates the NADPH oxidase that in turn induces the production of ROS (Li et al., 2014). flg22-FLS2 pathway can induce a transient elevation of cytosolic Ca^2+^ and the production of ROS, which ultimately induces stomatal closure to prevent bacterial invasion (Melotto et al., 2006). It is noteworthy that OPEN STOMATA 1 (OST1) and SLAC1 are essential factors for ABA-induced stomatal closure which have been also shown to mediate the flg22-regulation of stomatal closure. In fact, OST1 or second messengers (e.g. ROS or Ca^2+^) can activate SLAC1 to promote stomatal closure (Geiger et al., 2009; Vahisalu et al., 2010; Joshi-Saha et al., 2011). These findings suggest that there is crosstalk between ABA pathway and small peptide-mediated regulation of stomatal movement. In addition, MITOGEN-ACTIVATED PROTEIN KINASE MPK3/MPK6 are known to play roles in plant immune response (Mao et al., 2011; Meng et al., 2013; Su et al., 2017) and in small peptide-induced stomatal closure (Zhang et al., 2019).

Arabidopsis secreted peptide PIP1 can be recognized by RLK7, which is plasma membrane-localized LRR-RLK (Pitorre et al., 2010), and can amplify immunity response (Hou et al., 2014). A recent report indicates that PIP1 cooperates with salicylic acid to regulate stomatal immunity in *Arabidopsis thaliana* (Hou et al., 2019). In this study, we further confirmed that PIP1 could participate in the induction of stomatal closure. PIP1 could promote stomatal closure in a time- and dose-dependent manner. Moreover, anion channel SLAC1 played an important role in PIP1-RLK7 signaling pathway. We further showed that *BAK1*, *MPK3/6*, and an important signaling element of the ABA-pathway *OST1* were required for PIP1-induced stomatal closure. In addition, our research revealed that ROS and Ca^2+^ channels acted as the downstream components in PIP1-RLK7 signaling. Altogether, our study demonstrated the mechanism of PIP1-induced stomatal closure to some extent, and provided clues to delineate signal transduction pathways mediated by PIP1 in stomatal regulation and stress response.

## Materials and Methods

### Plant Materials and Growth Conditions

We used two wild type ecotypes of *A. thaliana* in this study: Columbia-0 (Col-0) and Landsberg-0 (Ler-0). Col-0 was the background of *PIP1* over-expression lines (PIP1-OE and PIP1-OE2), and mutants *rlk7* (*rlk7-2*, SALK_083114) (Hou et al., 2019), *bak1* (*bak1-4*, SALK_116202) (Hou et al., 2014), *bik1* (Zhang et al., 2010; Hou et al., 2014), *mpk3/mpk6* (SALK_151594/SALK_073907) (Xu et al., 2014; Zhang et al., 2019), *slac1-1*, *slac1-3*, and *rbohD/F* (CS9558) (Shen et al., 2017). But *ost1-1* and *ost1-2* were Ler-0 ecotype background. As for seedling growth, seeds were surface-sterilized with 75% ethanol for 3 min, then 95% ethanol for 1 min, and followed air-dried before use. The sterilized seeds were subsequently plated on half-strength Murashige and Skoog (1/2 MS) solidified medium (containing 1/2 MS salts, 1% w/v sucrose, and 0.7% w/v agar, pH 5.7) and then vernalized for 3 days at 4°C. After vernalization, seeds were transferred to a growth chamber (8-h light/16-h dark cycle, 100 µmol m^–2^ s^–1^ light, 70% relative humidity, a temperature regime of 22°C ± 2°C day/18°C ± 2°C night) for 1 week further growth. Then the seedlings were transplanted to pots containing soil mixture (vermiculite: rich soil, 1:2, v/v).

### Peptide Synthesis

According to the report (Hou et al., 2014), the peptide PIP1 used in this research was synthesized by Sangon Biotech Company (Shanghai, China) and the purity level of PIP1 was 98%. The sequence of peptide is shown from N terminus to C terminus as follows: RLASG-Hyp-SPRGPGH.

### Stomatal Closure Experiment

Stomatal apertures were measured as described previously with slight modification (Li et al., 2016). Fully expanded rosette leaves from about 4-week-old plants of every genotype were harvested for stomatal closure assay. Detached leaves were incubated in closure buffer (1 mM CaCl_2_, 20 mM KCl, 5 mM MES-KOH, pH 6.15) for 2.5 h in light. Then leaves were treated with PIP1, H_2_O_2_ or *Pst* DC3118 bacterial suspension (the final concentration of 10^8^ cfu/ml) for the indicated time, and non-bioactive PIP1 (hyperthermia inactivation) was the control of PIP1 treatment and water was used as the control of *Pst* DC3118 or H_2_O_2_. Subsequently, abaxial epidermal strips were peeled away by tweezers to make slides and placed on a light microscope (Olympus SZX16) to photograph randomly. The stomatal pore widths and lengths were measured using Image J (version: 1.37, https://imagej.nih.gov/ij/), and the stomatal aperture was calculated as the ratio of the inner pore width/pore length of each pair of stomata (Yang et al., 2017). All experiments were repeated for three independent biological replicates, and no less than 40 guard cells were measured for every sample. Statistical analyses were performed using a One‐way ANOVA followed by the significant difference test.

### Quantitative Real-Time PCR

The quantitative real-time PCR **(qPCR)** was used to analyze the transcript levels of *PIP1* and *SLAC1* in response to treatment. Briefly, *A. thaliana* 15-day-old seedlings grown on 1/2 MS solidified agar plates were transferred to 1/2 MS liquid medium for 24 h of incubation. Then 10 µM PIP1 or *Pst* DC3118 bacterial suspension (the final concentration of 10^8^ cfu/ml) was added to the medium and incubated for another hour. After indicated treatment time, seedlings were harvested and frozen quickly in liquid nitrogen. The transcription of *PIP1* was also identified by qPCR in the over-expression lines (PIP1-OE and PIP1-OE2) (Hou et al., 2014). Total RNA was isolated from seedlings using TRIzol reagent (Roche, Switzerland) and cDNA was synthesized using the Revert Aid First Strand cDNA Synthesis Kit (Thermo Fisher, USA). qPCR experiments were performed using the CFX96 Touch™ Real-Time PCR Detection system (Bio-Rad, Hercules, CA, USA) which based on SYBR Premix Ex Taq mix (Roche) with gene specific primers. The internal control was *ACTIN2*. All the quantitative analysis was repeated for three independent biological replicates. The primer sequences used are shown as follows: *ACTIN2* forward primer, 5′-GGTAACATTGTGCTCAGTGGTGG-3′, *ACTIN2* reverse primer, 5′-AACGACCTTAATCTTCATGCTGC-3′. *PIP1* forward primer, 5′-AATCGGGAGAATGGAAGTGC-3′, *PIP1* reverse primer, 5′-GACGCCAAACGCTGAAAC-3′. *SLAC1* forward primer, 5′-CCGGGCTCTAGCACTCA-3′, *SLAC1* reverse primer, 5′-TCAGTGATGCGACTCTT-3′.

### Guard Cell Isolation and Electrophysiology

Arabidopsis guard cell protoplasts were isolated according to Zhang et al. (2008) with some modifications. Briefly, the Arabidopsis abaxial epidermis were peeled from 12 to 14 expanded young leaves of 4-week-old plants. Then, all collected epidermis were blended in about 500 ml distilled water for 28 s and filtered through a 100-µm nylon mesh. Subsequently, the peels were transferred into 2 ml enzyme solution I (0.7% Cellulysin cellulase, 0.1% PVP-40, and 0.25% BSA in 55% basic solution (5 mM MES, 0.5 mM CaCl_2_, 0.5 mM MgCl_2_, 0.5 mM ascorbic acid, 10 µM KH_2_PO_4_, 0.55 M sorbitol, pH 5.5). The peels were digested in a shaking water bath at 80 rpm for 30 min at 25°C. Another 2-ml basic solution was added to enzyme solution I, and shaking was continued for a further 10 min. After that, the partially digested peels were filtered through a 100-µm nylon mesh and put into 2 ml of enzyme solution II, which contained 1.5% Onozuka cellulase RS, 0.02% cellulase Y-23, and 0.25% BSA in 100% basic solution. Then digestion of the peels continued by shaking at 60 rpm for at least 20 min. After digestion, these peels were collected and filtered through 30-µm nylon mesh. The guard cell protoplasts were obtained by centrifuging at 800 rpm for 5 min and washed twice by basic solution.

The whole-cell mode patch clamp experiment was performed as described previously (Pei et al., 1997; Pei et al., 2000; Wang et al., 2001; Acharya et al., 2013). To record the S-type anion channel currents, the bath solution contained 30 mM CsCl, 2 mM MgCl_2_, 1 mM CaCl_2_, and 10 mM MES (pH 5.6) and the pipette solution contained 150 mM CsCl, 2 mM MgCl_2_, 6.7 mM EGTA, 3.35 mM CaCl_2_, and 10 mM HEPES (pH 7.5). The osmolarity of the solutions was adjusted respectively with sorbitol to 480 and 500 mOsm for bath and pipette solutions. The ATP (Mg-ATP, 10 µM) and GTP (10 µM) were added to pipette solutions before use from stock solutions. To investigate PIP1 activation of Ca^2+^-permeable I_Ca_ channels, the pipette solution contained 10 mM BaCl_2_, 4 mM EGTA, 0.1 mM DTT, and 10 mM HEPES-Tris (pH 7.1). 5 mM NADPH was freshly added to the pipette solution before experiments. The bath solution contained 100 mM BaCl_2_, 0.1 mM DTT, and 10 mM MES-Tris (pH 5.6). Osmolarity was adjusted to 500 and 485 mOsm for the pipette solution and the bath solution respectively with D-sorbitol.

The anion channel currents were recorded using the Axopath-200B amplifier (Molecular Devices, Downingtown, PA, USA) after the whole-cell configuration was achieved. The holding potential was +30 mV, and voltage steps were applied from –145 to +35 mV in +30 mV increments, with a duration of 60 s for every test voltage. For PIP1 treatment, guard cell protoplasts were exposed to 10 µM PIP1 for 2 h before measurement and PIP1 was also added to both the bath and pipette solution. As to record Ca^2+^ channel currents, the whole-cell Ca^2+^ currents were recorded 5 min after achieving the whole-cell configuration. The holding potential was −13 mV and voltage ramps were from −200 to +80 mV. PIP1 or LaCl_3_ was added to the bath solution. To acquire and analyze the anion or Ca^2+^ channel currents, pCLAMP software (version 10.2; Axon Instruments, Sunnyvale, CA, USA) was used, and SigmaPlot 12.0 (Systat Software, Richmond, CA, USA) was used to draw the current-voltage plots and for data analysis.

## Results

### PIP1 Peptide Can Promote Stomatal Closure in *A. thaliana*

**Figure 1:**
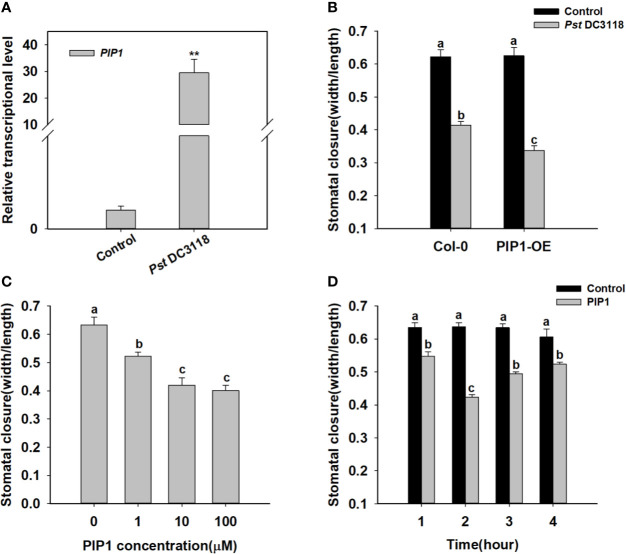
PIP1 induces stomatal closure in *Arabidopsis*. **(A)** Induction of transcript level of *PIP1* in response to *Pst* DC3118 treatment (the final concentration of 10^8^ cfu/ml). Asterisks indicate significant differences between means (P < 0.01). **(B)** Stomatal aperture in *PIP1* overexpression plants by treating with *Pst* DC3118 (the final concentration of 10^8^ cfu/ml). **(C)** Induction of stomatal closure by different concentrations of PIP1 peptides (0, 1, 10, 100 µM). **(D)** Time course of stomatal closure in response to 10 µM PIP1 treatment. In stomatal closure experiments, error bars indicate SE for three independent biological replicates. Different letters represent significant differences between groups using Holm-Sidak significant difference test after one-way ANOVA (P value < 0.05).

It is well known that stomata are the major entry sites for various pathogens, and PIP1 has been previously shown to function in plant immunity (Hou et al., 2014). Therefore, we found that exogenous treatment of *Pst* DC3118 bacteria, which is the COR deficient, could induce *PIP1* expression (**Figure 1**). Besides, *Pst* DC3118 could close the stomata in wild type, and the stomatal apertures were much smaller in *PIP1* over-expression lines than those of Col-0 (**Figure 1** and **Supplementary Figure S3**). These results indicated that PIP1 could close stomata to restrict pathogen entry *via* a PIP1-dependent manner. To further confirm the function of PIP1 in stomatal movement, we synthesized PIP1 peptides as described by Hou et al. (2014) and conducted stomatal closure experiments. Firstly, we analyzed stomatal aperture by exogenous application of different concentrations (0, 1, 10, 100 µM) of PIP1 peptides. The result showed that the stomatal aperture decreased in size under higher concentrations of PIP1 until the 10 µM concentration (**Figure 1**). Additionally, we also found that PIP1 could induce stomatal closure in a time-dependent manner. As shown in **Figure 1**, the stomatal aperture did not change obviously without PIP1 exposure but decreased within 1 to 2 h upon 10 µM PIP1 treatment, and the minimum aperture was observed at 2 h (**Figure 1**). Interestingly, the stomatal aperture began to recover at 3 h of PIP1 treatment. According to these results, we used 10 µM PIP1 as the optimal concentration and 2 h as the optimal treatment time for our follow-up experiments. These results indicate that secreted peptide PIP1 of *Arabidopsis* can play a role in regulating stomatal closure as a signaling molecule and can function in dose- and time-dependent manner.

### The Anion Channel SLAC1 Functions in PIP1-RLK7 Signaling

**Figure 2:**
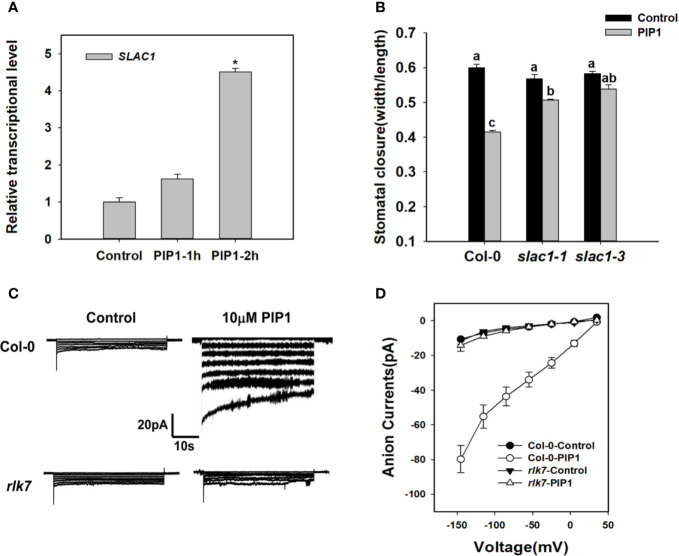
*SLAC1* plays an important role in PIP1-RLK7 signaling. **(A)** Evaluation of *SLAC1* induction in response to 10 µM PIP1 by qPCR. Asterisks indicate significant differences between means (*: P < 0.05). **(B)** Stomatal closure experiments in *slac1* mutants (*slac1-1* and *slac1-3*) in response to 10 µM PIP1. Error bars indicate SE for three independent biological replicates. Different letters represent significant differences between groups after one-way ANOVA (P value < 0.05). **(C)** Patch clamp whole-cell recordings of the anion currents in Col-0 and *rlk7* guard cell protoplasts with/without 10 µM PIP1. **(D)** Current/voltage relationships of whole-cell slow-type anion currents, as illustrated in **(C)**. The numbers of guard cells measured were as follows: Col-0-Control, n = 11; Col-0-PIP1, n = 7; *rlk7-*Control, n = 7; *rlk7*-PIP1, n = 7. Values are means ± SE.

Previous studies have shown that SLAC1 is the major component of S-type anion channels and plays an important role in ABA-, high CO_2_-, flagellin-induced stomatal closing responses in guard cells (Vahisalu et al., 2008; Kim et al., 2010; Guzel Deger et al., 2015; Zhang et al., 2018). These facts prompt us to find out whether *SLAC1* is also required for PIP1-induced stomatal closure. We found that exogenous PIP1 could improve the transcriptional level of *SLAC1* (**Figure 2**). In the presence of exogenous PIP1, the stomatal aperture reduced obviously in Col-0, but did not in *slac1* mutants: *slac1-1* and *slac1-3* (**Figure 2**). In addition, similar to PIP1, *Pst* DC3118 could induce the transcription of *SLAC1* (**Supplementary Figure S1**). Moreover, the genetic result showed that the stomatal closure of *slac1* mutants were hyposensitive to *Pst* DC3118 (**Supplementary Figure S1**). These findings suggest that *SLAC1* may play an important role in PIP1-induced stomatal closure and the response to *Pst* DC3118.

The research has shown that PIP1-induced stomatal closure is RLK7 dependent (Hou et al., 2019). To explore the mechanism of PIP1-RLK7 pathway in the regulation of stomatal response, we also conducted patch clamp whole-cell recordings of S-type anion currents in Col-0 and *rlk7* guard cell protoplasts under control condition and treated with 10 µM PIP1. The path clamp data showed that the size of anion currents of Col-0 guard cells increased when exposed to PIP1 (**Figure 2C**). However, the anion currents of guard cells from *rlk7* mutants failed to increase after addition of PIP1 (**Figure 2C**). In addition, *SLAC1* transcripts did not change in *rlk7* with PIP1 treatment (**Supplementary Figure S2**), which suggest RLK7 is essential for PIP1 induced *SLAC1* transcription, and thus to facilitate the anion solutes efflux of guard cells to close stomata.

### *BAK1*, *MPK3/6*, and *OST1* Take Part in PIP1-Induced Stomatal Closure

**Figure 3:**
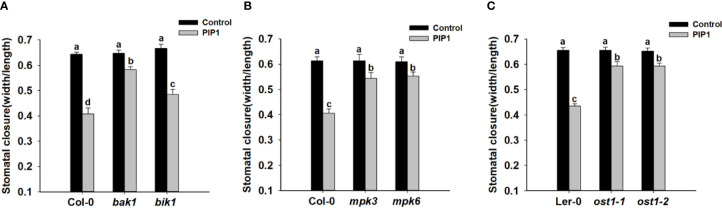
*BAK1*, *MPK3*, *MPK6*, and *OST1* are involved in PIP1-induced stomatal closure. **(A–C)** 10 µM PIP1-induced stomatal closure in *bak1* and *bik1* mutants **(A)**, *mpk3* and *mpk6* mutants **(B)**, *ost1-1* and *ost1-2* mutants **(C)**. Values are means ± SE (n = 3). All the experiments were performed in three independent biological replicates with similar results. Different letters indicate significant difference between groups after one-way ANOVA (P value < 0.05).

The receptor kinase BAK1 and BIK1 often play an important role in peptide signaling or immune response by forming heteromeric co-receptor complexes with multiple LRR-RLK receptors (such as FLS2, PEPR1, and so on) (Chinchilla et al., 2007; Lu et al., 2010; Liu et al., 2013; Zheng et al., 2018). Additionally, BAK1 can regulate ABA-induced stomatal closure in guard cells (Shang et al., 2016). Previous report indicates that PIP1-RLK7 signaling is partially dependent on *BAK1*, but independent of *BIK1* (Hou et al., 2014). Therefore, we would like to test the possible functions of *BAK1* and *BIK1* in PIP1-induced stomatal closure. Firstly, we used genetic approach to further examine if *BAK1* and *BIK1* participated in PIP1-induced stomatal closure. The stomatal closure experiments of Col-0, *bak1*, and *bik1* showed that the stomatal aperture of *bak1* mutants became insensitive to PIP1 when compared with Col-0 and *bik1* (**Figure 3**). Moreover, consistent with the observing result in *rlk7*, further data revealed that PIP1 could not activate *SLAC1* transcription in mutant *bak1* (**Supplementary Figure S2**), however, PIP1 could normally induce *SLAC1* transcription in mutant *bik1* (**Supplementary Figure S2**). Our findings suggest that *BAK1* instead of *BIK1* may be required for PIP1-induced stomatal closure.

Published reports show that ﬂg22-induced stomatal closure is compromised in *mpk3* and *mpk6* mutants (Montillet et al., 2013) and *MPK3/MPK6* mediate peptide-induced stomatal movement (Zhang et al., 2018). Since MPK3 and MPK6 have been shown to be activated upon PIP1 induction in Arabidopsis seedlings (Hou et al., 2014), thus we also tested the contributions of *MPK3* and *MPK6* to PIP1-induced stomatal closure. The result showed that PIP1-triggered stomatal closure was impaired in both *mpk3* and *mpk6* mutants (**Figure 3**). Our finding indicate that *MPK3* and *MPK6* also serve as the downstream components in PIP1-RLK7 pathway.

Stomatal movement is regulated by the phytohormone abscisic acid (ABA) (Lee and Luan, 2012). The ABA-triggered activation of guard cell anion channels results in the efflux of anions, which in turn promotes guard cells to close the stomata (Wang et al., 2013). In addition, we has established that *SLAC1* plays a role in stomatal response in PIP1-RLK7 pathway. Therefore, we would like to explore the role of ABA signaling in PIP1-induced stomatal closure in guard cells. OST1, a SnRK2-type kinase, which has been shown to be critical signaling element for ABA-induced activation of S-type anion channels and ABA-induced stomatal closure (Joshi-Saha et al., 2011; Brandt et al., 2012). OST1 also regulates flg22-induced stomatal closure and the activation of S-type anion channels (Guzel Deger et al., 2015). These facts prompted us to examine the possible function of *OST1* in PIP1-induced stomatal closure. The result showed that the stomatal apertures of *OST1* mutants *ost1-1* and *ost1-2* were hyposensitive to PIP1 when compared with wild-type Ler-0 (**Figure 3**). Therefore, *OST1* may also be required for PIP1-induced stomatal closure and *OST1* may be a common node of PIP1 signaling and ABA signaling pathway in stomatal regulation.

### ROS Production and Ca^2+^ Signaling Are Required for PIP1-Regulated Stomatal Closure

**Figure 4:**
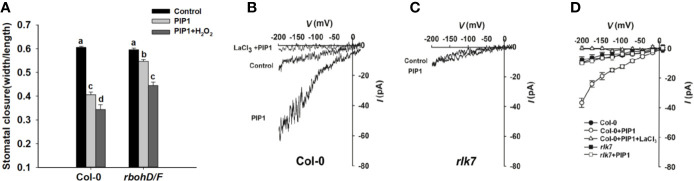
ROS and Ca^2+^ signaling participate in PIP1-regulated stomatal closure. **(A)** The stomatal closure in Col-0 and double mutant *rbohD/F* exposed to 10 µM PIP1 or 10 µM PIP1+100 µM H_2_O_2._ Error bars indicate SE for three independent biological replicates. Different letters represent significant differences between groups after one-way ANOVA (P value < 0.05). **(B, C)** Typical whole cell recordings of Ca^2+^ currents in guard cell protoplasts isolated from Col-0 **(B)** and *rlk7* **(C)** with 10 µM PIP1, 10 µM PIP1+1 mM LaCl_3_. **(D)** Current/voltage curves of time-activated Ca^2+^ currents as indicated in **(B, C)**. The number of guard cells measured as follows: Col-0 (control): 5; Col-0 (PIP1): 6; Col-0 (PIP1+LaCl_3_): 6; *rlk7* (control): 5; *rlk7* (PIP1): 6.

ROS, crucial signal molecules, participate in the regulation of stomatal movement (Purohit et al., 1994; Murata et al., 2015). PIP1 also has been reported to induce ROS production in adult leaves of *Arabidopsis* (Hou et al., 2014; Hou et al., 2019). AtRbohD and AtRbohF play essential roles in ROS production. Therefore, we genetically examined the stomatal response to PIP1 in *rbohD/F* double mutants, which fail to produce ROS. The result showed that *rbohD/F* double mutants were impaired in PIP1-induced stomatal closure but stomatal aperture decreased again when treated with additional H_2_O_2_ (a form of ROS) (**Figure 4**). The result suggest that ROS is required for PIP1-induced stomatal closure.

Ca^2+^ also functions as a key second messenger, which participates in the regulation of stomatal closure (Moscatiello et al., 2006) and Kwak et al. (2003) have reported that ROS can activate Ca^2+^-permeable channels and can produce concurrent cytosolic Ca^2+^ increase. In this study, we found that the ROS production participated in PIP1-induced stomatal closure, and it was needed to analyze whether Ca^2+^ also could be induced by PIP1. By using patch clamp, we detected a clear increase of Ca^2+^ currents through plasma membrane into cytoplasm after treatment with PIP1, but no obvious change of Ca^2+^ currents under control conditions was observed in guard cells (**Figure 4B**). The application of LaCl_3_, a calcium channel blocker, completely abolished the PIP1-induced Ca^2+^ influx (**Figure 4B**), indicating PIP1 can stimulate Ca^2+^ influx through activating the guard cell Ca^2+^ channels. In addition, our finding indicated that RLK7, receptor of PIP1, was required for the PIP1-activated Ca^2+^ currents (**Figure 4C**).

## Discussion

Plant stomata, consisting of a pair of guard cells, are dynamic structures that open or close to modulate gas exchange and water loss, and also allow plants to respond appropriately to diverse pathogens invasion. Therefore, stomata play essential roles in abiotic and biotic stress responses (Blatt et al., 2017). The regulation of stomatal movement is complex, and as time progresses, researchers discover new signaling elements which make the signaling networks of stomatal movement more complex (Chen et al., 2019; Sun et al., 2019). Recent years, in addition to plant growth and development, plant small signaling peptides have been implicated in stomatal aperture regulation (Li et al., 2014; Zheng et al., 2018; Qu et al., 2019). The Arabidopsis secreted peptide PIP1 plays a role in plant immune response, and the function of PIP1 is receptor RLK7-dependent (Hou et al., 2014). The PIP1-RLK7 pathway also functions with salicylic acid to regulate stomatal immunity (Hou et al., 2019). However, it is still unclear that the specific mechanism of PIP1-RLK7 signaling in stomatal regulation. In this study, we further identify new factors which play critical roles in PIP1-induced stomatal closure and the possible mechanism by which PIP1 regulates stomatal movement. This study also contributes to combine peptide-signaling with stomatal signal transduction pathway in guard cells.

We firstly further explored the impact of PIP1 on stomatal closure. Our findings showed that PIP1 could induce stomatal closure in a dose-dependent and a time-dependent manner (**Figure 1C**). The elevated transcription of *PIP1* induced by *Pst* DC3118 (**Figure 1**) and the much smaller stomatal aperture of PIP1-OE lines exposed to *Pst* DC3118 (**Figure 1** and **Supplementary Figure S3**), suggest that PIP1 can mimic the function of *Pst* DC3118 and can resist the pathogenic aggression through promoting stomatal closure. In addition, RLK7 acts as a PIP1 receptor in guard cells that activates stomatal immunity response upon PIP1 detection (Hou et al., 2019). Therefore, we further explored the mechanism of PIP1-RLK7 pathway induced stomatal closure. It is well known that the activation of S-type anion channels plays an important role in decreasing guard cell turgor and then leading to stomatal closure (Kim et al., 2010; Zhang et al., 2018; Munemasa et al., 2019). We found that SLAC1, an important S-type anion channel, mediated the PIP1-RLK7 signaling in regulating stomatal closure. The transcriptional level of *SLAC1* was enhanced by treating with PIP1 (**Figure 2**) and the stomatal apertures of *slac1-1* and *slac1-3* mutants failed to respond to PIP1 (**Figure 2**). To obtain more concrete evidences for the involvement of *SLAC1* in PIP1-induced stomatal closure, we found that *SLAC1* transcripts did not change in mutant *rlk7* with PIP1 treatment (**Supplementary Figure S2**). Additionally, we also tested the S-type anion currents in Col-0 guard cell protoplasts and found that PIP1 increased the size of anion currents (**Figure 2C**). However, PIP1 could not activate the S-type anion channels in *rlk7* mutants (**Figure 2C**). The results confirm that PIP1-RLK7 signaling induce stomatal closure through the activation of S-type anion channel SLAC1.

In this study we has established that *SLAC1* plays an important role in stomatal response to PIP1-RLK7 pathway, then we further explored the signaling mechanism for the activation of SLAC1 and sought key factors which transmitted the signal from PIP1-RLK7 to SLAC1. BAK1 belongs to the SERK family and acts as forming ligand-induced heteromers with multiple LRR-RLKs, including X subfamily of LRR-RLK BRI1 and XII subfamily of LRR-RLK FLS2 (Chinchilla et al., 2007; Wang et al., 2008). Previous study has found that PIP1-induced root inhibition is less predominant in *bak1* mutants (Hou et al., 2014). Here, we also found that *bak1* was less sensitive to PIP1 for the induction of stomatal closure (**Figure 3**) and PIP1 could not induce the expression of *SLAC1* in *bak1*, which was similar to that in *rlk7* (**Supplementary Figure S2**). These results suggest that *BAK1* may be required for PIP1-RLK7 signaling in inducing stomatal closure process. In future research, it will be necessary to determine whether BAK1 serves as a co-receptor of RLK7 in perceiving the PIP1 ligand. In plants, MPKs widely function in various processes of plant development or in response to biotic and abiotic stresses (Rodriguez et al., 2010; Lee et al., 2016). Arabidopsis genome encodes 20 MPKs and among these, MPK3 and MPK6 function in flg22-induced stomatal closure (Montillet et al., 2013). In addition, MPK3 and MPK6 are involved in stomatal immunity and MPK3/MPK6 cascade-induced stomatal closure is related to malate/citrate metabolism (Su et al., 2017). Recently, it has been shown that MPK3 and MPK6 also serve as the downstream components in peptide CLE9-induced stomatal closure (Zhang et al., 2019). PIP1 also can induce the phosphorylation of MPK3 and MPK6 in an RLK7-dependent manner (Hou et al., 2014). In our study, we demonstrated that both mutant *mpk3* and *mpk6* were insensitive to PIP1 (**Figure 3**), which indicates that PIP1-RLK7 possibly stimulates stomatal closure through the activation of *MPK3/MPK6*-mediated pathways. But it is required to further investigate how PIP1-RLK7 influences *MPK3/MPK6* and then induces stomatal closure.

There are multiple signaling elements have been discovered which play critical roles for ABA-induced stomatal closure. We hypothesize that some key signaling elements may also play roles in PIP1-induced activation of S-type anion channels. Therefore, we examined the possible functions of several signaling molecules including a SnRK2-type kinase, OST1. *OST1* has been demonstrated to contribute in flg22-FLS2 signaling, and it is required to activate the guard cell S-type anion channels to induce stomatal closure (Melotto et al., 2006; Koers et al., 2011; Ye et al., 2015). Therefore, we expected that PIP1-RLK7 signaling may also require *OST1* to initiate stomatal closure. The stomatal experiment result indicated that, to some extent, *OST1* mediated the regulation of PIP1-induced stomatal closure (**Figure 3**). Our finding suggests that PIP1-mediated stomatal response and ABA-induced stomatal closure overlap in common downstream components including *OST1*. BAK1 can serve as an upstream activator of OST1 and can form a complex with OST1 in response to ABA in guard cells (Shang et al., 2016). In addition, *BAK1* has been shown to play a role in PIP1-RLK7 signaling. Accordingly, we assume that BAK1 may also function by interacting with OST1 in PIP1-RLK7 pathway which is similar to in ABA-signaling. In future study, we would like to confirm the relationship between *BAK1* and *OST1* in PIP1 signaling. Furthermore, ROS have been considered as crucial secondary messages in ABA-induced stomatal closure and OST1 catalyzes ROS production (H_2_O_2_) mediated by NADPH oxidase (RbohD and RbohF) (Brandt et al., 2012). In our study, we detected double mutant *rbohD/F* was insensitive to PIP1 for closing stomata (**Figure 4**). The data suggest that ROS generation is required for PIP1-induced stomatal closure. Besides, the study (Kwak et al., 2003) has been reported that H_2_O_2_ application in guard cells activates Ca^2+^ channels and produces concurrent cytosolic Ca^2+^ increase. Here, the results indicated that PIP1 could also activate Ca^2+^-permeable channels in RLK7-dependent manner (**Figures 4B–D**), suggesting that Ca^2+^ signal may participate in PIP1-induced stomatal closure.

**Figure 5:**
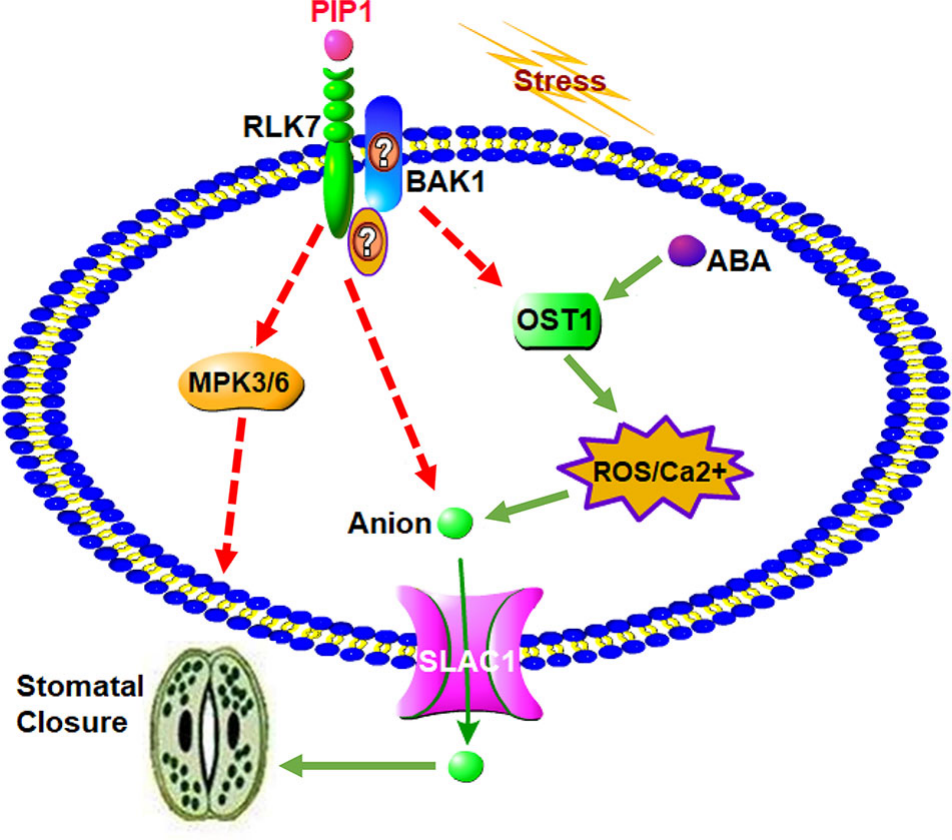
A proposed model for the role of PIP1 in the regulation of stomatal closure. PIP1 can bind to its receptor RLK7, then activates S-type anion channel SLAC1 to induce stomatal closure. PIP1-RLK7 also possibly recruits the coreceptor BAK1 or other proteins to form a receptor complex, and *OST1* may act as downstream factor to facilitate ROS and Ca^2+^ signaling to close stomata. In addition, *MPK3* and *MPK6* are also required for PIP1 regulating stomatal movement signaling in guard cells.

In conclusion, a possible working model for the role of secreted peptide PIP1 in the regulation of stomatal closure is presented in **Figure 5**. The function of PIP1-induced stomatal closure depends on the perception by its receptor RLK7, then further activates S-type anion channel SLAC1 to lead to stomatal closure. On one hand, RLK7 may share a downstream component, OST1, with the well-studied guard cell ABA pathway by recruiting BAK1 or other unknown factors. OST1 can induce the production of ROS, and ROS further activate Ca^2+^ channels. These factors ultimately result in the activation of anion channel SLAC1 to promote stomatal closure. On the other hand, we found that PIP1-mediated regulation of stomatal aperture requires *MPK3/MPK6*, which serve as downstream mediators. Collectively, our study further reveals that PIP1 peptides can regulate stomatal aperture and explains the possible functional mechanism. It not only connects peptide signaling with stomatal regulation, but also illustrates that PIP1 may function in stress response by closing stomata. Nevertheless, there are many specific questions remaining to be answered and further work will be carried out to clarify molecular mechanism of PIP1-induced stomatal closure. We also need to further investigate how PIP1 influences *MPK3/MPK6* pathway and the relationship between RLK7 and BAK1 will be confirmed by protein-protein interaction approaches (such as Co-IP). In addition, we cannot confirm if OST1 mediates PIP1-signaling in BAK1-dependent manner, or there are other factors participating in signal transduction from PIP1 perception to the activation of SLAC1. We would like to study in future by employing multipronged approaches.

## Data Availability Statement

All datasets generated for this study are included in the article/**Supplementary Material**.

## Author Contributions

WZ and DC are corresponding authors. JS and WD designed and performed the major experiments. JS analyzed the results and wrote the manuscript. LZ performed few experiments and interpreted the data. MW, BA, DC, and XZ designed a few experiments and analyzed some data. WZ conceived and designed the study, interpreted the results, and corrected the manuscript. All authors contributed to the article and approved the submitted version.

## Funding

This work was financially supported by Distinguished Young Scholar of Shandong University (61200088963137), Natural Science Foundation of Shandong Province (ZR201807100168) and Cooperation project on nutritional value and health care function of special vegetable for pregnant women and infants (61200012001902).

## Conflict of Interest

The authors declare that the research was conducted in the absence of any commercial or financial relationships that could be construed as a potential conflict of interest.

## Supplementary Material

Figure S1 **(A)** The *Pst* DC3118 induction on *SLAC1* transcription in Col-0 seedlings. Asterisks indicate significant differences between means (*: P < 0.05). **(B)** Stomatal closure experiments in *slac1-1* and *slac1-3* mutants in response to *Pst* DC3118 (the final concentration of 10^8^ cfu/ml). Error bars indicate SE for three independent biological replicates. Different letters represent significant differences between groups after one-way ANOVA (*P* value < 0.05).

Figure S2 The *SLAC1* transcription in Col-0 and *rlk7*, *bak1*, *bik1* mutants upon PIP1 treatment. Asterisks indicate significant differences between means (* *P* < 0.05).

Figure S3
**(A)** The qPCR-based assessment of *PIP1* transcription in Col-0 and the over-expression lines PIP1-OE and PIP1-OE2. **(B)** Stomatal aperture in PIP1-OE2 by treating with *Pst* DC3118 (the final concentration of 10^8^ cfu/ml). Different letters represent significant differences between groups after one-way ANOVA (P value < 0.05).
