# Supplementary figures and images for: Secreted Peptide PIP1 Induces Stomatal Closure by Activation of Guard Cell Anion Channels in Arabidopsis
Source: Front Plant Sci. 2020 Jul 8;11:1029. doi: 10.3389/fpls.2020.01029 (PMC7360795; doi:10.3389/fpls.2020.01029)

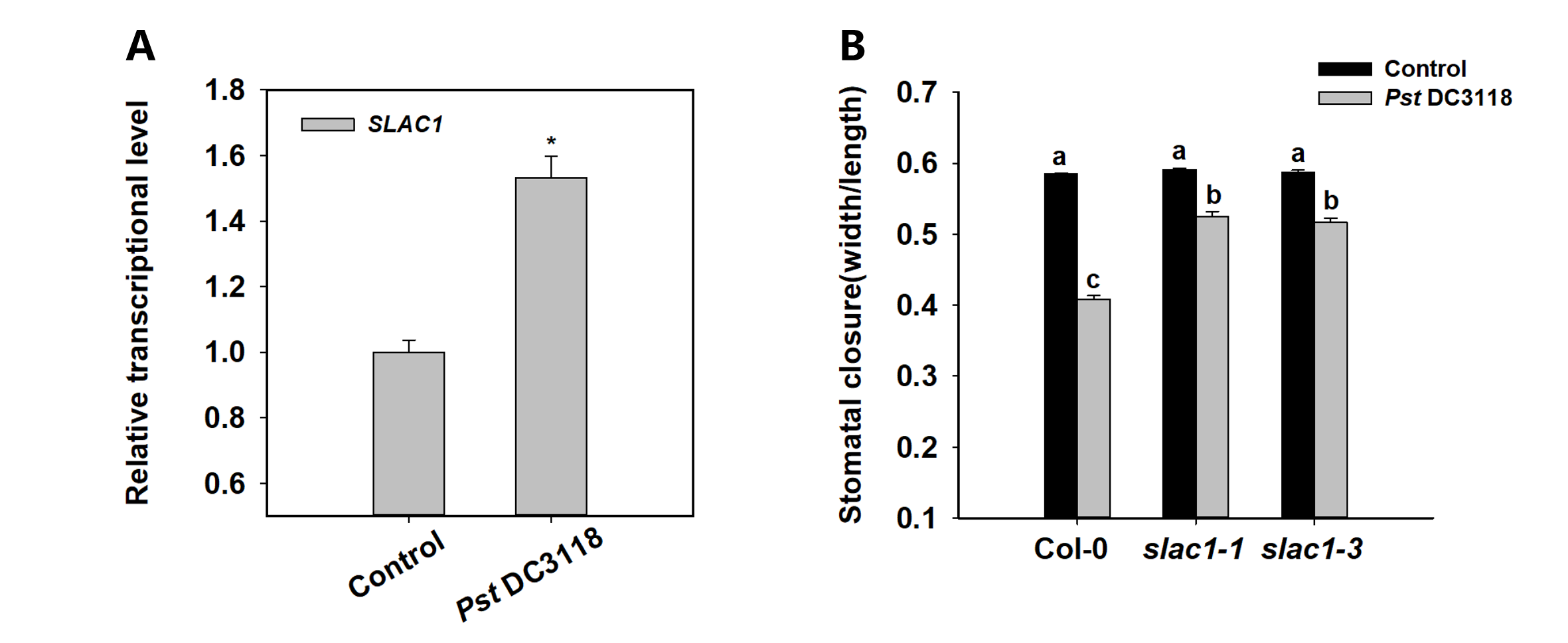

Supplement: Figure S1 — (A) The Pst DC3118 induction on SLAC1 transcription in Col-0 seedlings. Asterisks indicate significant differences between means (*: P < 0.05). (B) Stomatal closure experiments in slac1-1 and slac1-3 mutants in response to Pst DC3118 (the final concentration of 108 cfu/ml). Error bars indicate SE for three independent biological replicates. Different letters represent significant differences between groups after one-way ANOVA (P value < 0.05). [file Image_1.tif]

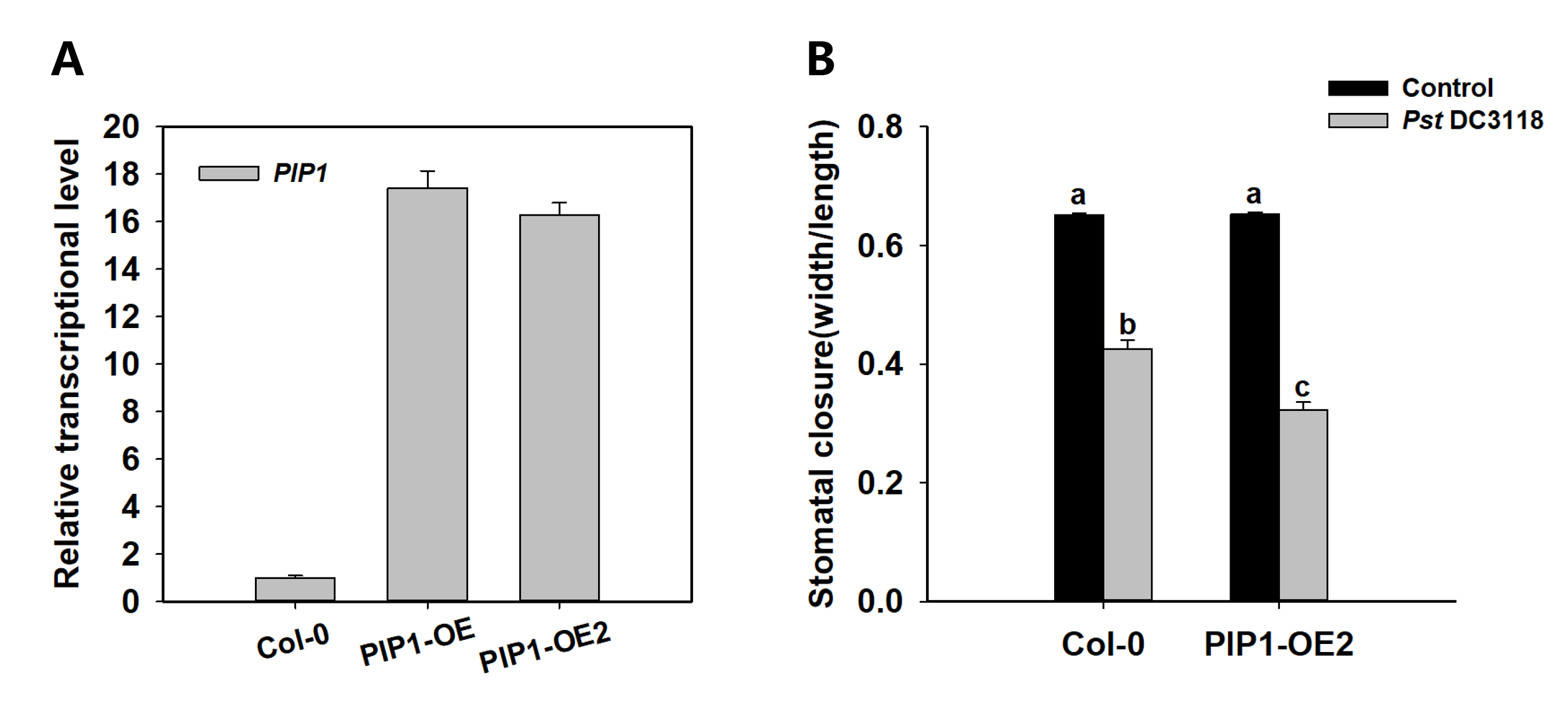

Supplement: Figure S3 — (A) The qPCR-based assessment of PIP1 transcription in Col-0 and the over-expression lines PIP1-OE and PIP1-OE2. (B) Stomatal aperture in PIP1-OE2 by treating with Pst DC3118 (the final concentration of 108 cfu/ml). Different letters represent significant differences between groups after one-way ANOVA (P value < 0.05). [file Image_3.tif]
